# Primary healthcare services and maternal mortality in Ugep

**DOI:** 10.1016/j.amsu.2021.102691

**Published:** 2021-08-06

**Authors:** Ogadimma Arisukwu, Stephen Akinfenwa, Chisaa Igbolekwu

**Affiliations:** a SDG 10, Nigeria; b Department of Sociology, Landmark University, Nigeria

**Keywords:** Maternal health, Mortality, Community partnership, Pregnancy, Childbearing

## Abstract

This paper focusses on pregnancy related deaths which is a contemporary issue in modern day Nigeria. Maternal Mortality is more pronounced in Ugep, Cross River State with the maternal mortality ratio of l200/100,000 which is higher than the national figure of 1100/100,000 (Nigerian Partnership for Safe Motherhood, 2018). In Nigeria, about 75 % of women die as a result of these complications either during the course of giving birth or the week preceding delivery (Choudhry, 2012). Recent statistics shows that Maternal deaths account for 32% of all deaths among women age 15–49 in Nigeria. The maternal mortality rate for the seven-year period preceding NDHS 2013 survey was 1.05 maternal deaths per 1000 women. The maternal mortality ratio was 576 maternal deaths per 100,000 live births. The lifetime risk of maternal death indicates that out of every 30 women in Nigeria, one will have a death related to pregnancy or childbearing (NDHS, 2013). Statistics further revealed that pregnancy complications in 2012 led to the death of over 52,000 women in Nigeria (Dada, 2016). Maternal Mortality in the seven years preceding the National Demographic Health Survey in 2013 records the figure of live births to be 575 per 100,000, which implies that for one single pregnancy complications resulting to death, more than 20 others are confronted with disabilities which may last a life time, (NDHS, 2013). The theoretical application combines aspects of the Environmental Precedence Theory, Rational Choice Theory and the Health Belief Model (HBM) to explain core variables of the relationship between maternal health practices and pregnancy outcomes. Several conclusions were inferred from the application of sociological theories to the chosen contemporary issue.

## 1 Introduction

Contemporary issues in modern day Nigeria can be best understood through the application of sociological theories. There is an intricate relationship between theory and research. No discipline can develop without an appropriate mix between theory construction and empirical investigation. Research being the investigation of the relationship implied by the theory or accumulation of views by explain it and illuminating its relationship with other phenomena. According to Ref. [1], science aims to organize and condense existing knowledge, with the objective to give explanations about observed events as well as the relationship between events on the basis of explanatory principles embodied in theory. Thus, the aim of science is to describe, explain, and establish causation and relationships and predict. These objectives entail a symbolic relationship between theory and research, research and theory are interdependent aspects of the sociological enterprise rather than distinctive and mutually exclusive activities. No wonder the relationship between theory and research is that of symbolism. According to Ref. [2], the theory gives an organized and systematic explanation of observed facts and states laws that demonstrate the relatedness to a particular aspect of life. A theory is also seen as a formal logic deductive system which involves a set of assumptions which form the basis for the derivation of variable explanations and deductions.

In this study, an attempt will be made to review different theoretical orientations with a view to explaining their relevance to the understanding of maternal health and maternal mortality in Nigeria. During the theoretical review, the adequacy of each of the theories to be considered will be highlighted. There are several theoretical explanations relating to the Utilization of Maternal Healthcare Services and Maternal Mortality in Ugep. Some of these theories include the Health Belief Model [3], Social Determinants of Health Theory, and Symbolic Interactionism Theory [4].

The above-mentioned theories will be critically examined, with a view to finding their relevance to the topic under study. Out of the above-mentioned theories, the researcher will adopt a particular one or a combination of theories to be used in the study on the utilization of Maternal Healthcare Services and Maternal Mortality in Ugep. The reason for this decision is not unconnected with the inadequacy of some of the mentioned theories. Not all theories can adequately explain the utilization of Maternal Healthcare Services and Maternal Mortality among the people of the research setting. Secondly, by adopting one of the theories, the researcher will be more focused and have a direction, since the adequacy and inadequacy of all theories will be discussed with a view to finding the one that is most suitable to this study. The first five theories that will be discussed have a direct impact on the utilization of Maternal Healthcare Services and Maternal Mortality. While the other theories are Sociological Theories that can be adopted in the study. However, before a detailed discussion of the theory, a brief discussion of the proposed study “Utilization of Maternal Healthcare Services and Maternal Mortality in Ugep” shall be done.

## 2 Brief discussion of the contemporary issue “Utilization of Maternal Healthcare Services and Maternal Mortality in Ugep"

### 2.1 Background to the study

Maternal care Services (MHS) are not unconnected with reducing maternal mortality as well as infant and neonatal health outcomes [5]. Ante-Natal Care provides pregnant women with relevant information and risks aimed at enhancing their utilization of MHS [6]. Complications are known to be directly or indirectly connected to maternal death, about 75 % of women die as a result of these complications either during giving birth or the week preceding delivery [7] It is therefore necessary that skilled birth attendants attend to pregnant women and these skilled birth attendants include doctors, community health officers, nurses, etc [8]. Recent statistics show that pregnancy complications in 2007 led to the death of over 52,000 women in Nigeria [9]. For seven years prior to the National Demographic Health Survey in 2008, maternal health experienced a precipitous decline, records show the figure of live births to be 545 per 100,000, which implies that for one single pregnancy complication resulting in death, more than 20 others are confronted with disabilities which may last a life time [9].

Women in Bikobiko, Ijiman, Ijom, Ikpakapit, and Ketabebe, representing the five traditional councils in Ugep of Yakurr Local Government Area, die during pregnancy and childbirth especially early postpartum. What it means is that unskilled birth attendants are still being patronized despite the ante-natal care received from skilled obstetric service deliveries. In developing countries such as Nigeria, pregnant women are apprehensive of child birth, as pain and death are perceived as strong possibilities during child birth, this situation is very unlike what obtains in the developed world [10]. Study has shown that there exist a link between the socio-economic status of women and utilization of maternal health services, [11]. Pregnant women tend not to have trust in the modern healthcare infrastructure to reduce this apprehension is regrettably low and for some pregnant women, the patronage of traditional birth attendants for delivery is the way out of this predicament [12]. Health personnel attended to lower proportions of delivery as it depleted from 43 % in 1990 to 38.9 % in 2008 [13]. Although by 2012, the figure increased to 53.6 %, notwithstanding this increment, the trend is on an unenviable decline, with interventions that have been put in place not achieving the desired objectives [12]. Maternal health is the total wellbeing of the woman and it cuts across physical wellbeing either during pregnancy, childbirth, and even the period of postpartum [14,15]. Since the late 1980s, maternal death have been a central topic of several international summits, culminating in the Millennium Summit which held in the year 2000 [16]. The agreement at the summit was that there should be an improvement in reproductive health owing to the fact that it plays an important role in maternal wellbeing with the emphasis that women should be empowered and adequately informed on the need to patronize appropriate and acceptable healthcare facilities which should be affordable, effective and permit unhindered access. With these in place, pregnant mothers will be able to safely carry the pregnancy to parturition with a highly minimized rate of maternal mortality and increased rates of infant survivability [17].

### 2.2 Statement of the problem

Pregnancy poses a threat to the life of women and this is so most especially in developed countries. Study shows that 23% of maternal mortalities in the World take place in Nigeria [18]. It is equally important to get the views and experiences of women with regards to maternal health services use and its impact on their wellbeing [19]. This threat to the lives of women is a constraint on their life expectancy. The veritable index with which the wellbeing of women is measured in any country is the maternal mortality rate [20], and this wellbeing depends on how well they access and use maternal healthcare services. In Cross River state, the baseline survey conducted in 2010 indicated that Maternal Mortality rate was 545/1000, 100,000 live births representing the mortality ratio in 2008 [21]. When discussion center on maternal mortality, the emphasis by scholars has always been on the medical causes of maternal mortality. With the emphasis on medical causes, it means malfunctions of the body and symptoms were given the bulk of attention without adequate concentration on the context within which maternal healthcare impacts the lives of women at all stages of pregnancy. Many studies, such as [[22], [23], [24], [25]], have only assessed the medical proximate determinants of maternal mortality, but the contexts that precipitates the utilization of maternal healthcare services and maternal mortality have not been adequately examined.

In the face of all these situations, there has been no convergence of opinions among researchers on a holistic and encompassing analysis of the situation of maternal health. This is because some of the issues are looked upon as private (Micro-level and household) while others are treated as public (Macro-level and community). Both of them, however, are an integral parts of activities that influence maternal health, but nevertheless, analytical work in this area is very limited [26].

Although significant, anthropological and demographic research has been conducted among the Yakurr by Forde and Obono. Forde drew early scholarly attention to Yakurr studies while Obono presented studies on sociocultural contexts of Yakurr fertility, however, the implications of fertility on pregnant mothers, culminating in the utilization of maternal healthcare services through ante-natal, natal and postnatal maternal care were not fully worked out in these epic studies. Furthermore, publications on maternal health in Nigeria have addressed the issues in other areas to the almost exclusion of Ugep, it is against this background that this study examines the utilization of maternal healthcare services and maternal mortality in Ugep, Yakurr Local Government Area.

### 2.3 Study objective

The general objective of this paper is to apply sociological theories to the contemporary issue of Maternal Healthcare Services and Maternal Mortality in Ugep, Yakurr Local Government Area of Cross River state, Nigeria.

## 3 Sociological theories applied to the Utilization of Maternal Healthcare Services and Maternal Mortality in Ugep

### 3.1 social action theory

Max Weber proposed the Social Action Theory in (1922) and the theory of social action looks at the reaction between a stimulus and the ultimate response [27]. Max Weber, a German Sociologist (1864–1920) in his action theory, tried to analyze the action of individuals in typical situations, he saw the need to understand and interpret human behavior based on the meanings behind their actions that is, understanding human behavior and the reasons behind such behaviors.

Verstehen is the way adopted by Weber to interpret motives. An empathetic understanding is also known for verstehen. In this case, one can understand a person's motives better by trying to wear shoes. Thus, the utilization of maternal healthcare services and maternal mortality experience can better be understood if one empathizes and thus will be able to influence one's choice on the utilization of maternal healthcare services [28].

Max Weber further explained that all human actions are directed by meanings, which led to his identification of various types of actions that are distinguished by the subjective meanings given to them. Four major types of social action were distinguished by Weber: purposeful or goal-oriented rational action (zweckrational) in which both goals and means are rationally chosen, value-oriented (wertrational) which is characterized by striving for a substantive goal, which in itself may not be rational, emotional or affective motivations which is hinged on the state of emotion of the actor and finally, traditional action which is guided by customary behaviour through reliance on the laid down customs, beliefs and values. The primary concern of Weber was with western society and he opined that behaviour had come to be influenced by goal-oriented rationality rather than by tradition as it used to be [28].

## 4 Weaknesses and application of social action theory to utilization of maternal healthcare services and maternal mortality among the Ugep of Southern Nigeria

The application of Weberian Social Action theory helps to understand the subjective meanings that the pregnant woman attaches to maternal healthcare services. Interactionist sociologists believe that social structures and institutions are not the primary drivers or determinants of social behaviour. People play a much proactive role in shaping their social life. The pregnant woman plays active roles in determining if she would present for maternal healthcare services or not. Most people engage in voluntary behaviour because they have free will. Maternal healthcare services and maternal mortality among women in Ugep is based on the free will of women to utilize maternal healthcare services.

The social action theory advanced that although people operate as individuals, nevertheless the attitudes and actions of other people around them influence the way they think and the way they behave at the household, individual, and community level. When there is a high use of maternal healthcare services, mothers will be influenced to use ante-natal, natal, and post-natal healthcare services. People acquire knowledge about appropriate behaviour in particular situations, social responses are elicited in particular contexts. The pregnant mother has learnt what constitutes appropriate behaviour on the use or nonutilization of maternal healthcare services.

Community level factors through symbolic interaction teaches what constitutes appropriate maternal health behaviour in a community where emphasis is on the use of ante-natal, natal, and post-natal maternal healthcare services. Pregnant women are more likely to make themselves available for maternal healthcare services. The direction of use of maternal healthcare services in a community is also an important indicator of how pregnant women would use maternal healthcare services, if the emphasis at the household or community level is on the preference of local birth attendants, the tendency for expectant women to patronize traditional birth attendants becomes high.

Society evolves when people interact in social groups and make sense of each other behaviour, role and norms exist in all societies, but they are flexible guidelines rather than unchangeable frameworks over which we have no control, whatever the role an individual plays in the society is open to negotiation and individual interpretation. The pregnant women can have acquired a social entities in the process of socialization, the socialization shows what the society expects those in a particular role to live up to. Pregnant mothers are expected to utilize maternal healthcare services in some socio-cultural environment, while in others, a social identity of utilization of Maternal Healthcare Services may not be important.

The labeling perspective under the broad social action theory believes that there is no such thing as a right or wrong act to classify an act as deviant, the dominant group in the socio-cultural environment must label and define the act as deviant, if the use of maternal healthcare services is classified as deviant, pregnant women may not be willing to patronize maternal healthcare services which will increase the rate of maternal mortality in the socio-cultural environment.

The challenges in the application of social action theory in the use of maternal healthcare services and maternal mortality among the Ugep of south-south Nigeria are that the theory pays less attention to the structures of society, structures such as gender and ethnicity as well as social class tend to serve as constraints on individual behaviour and the theory is considered overly deterministic.

## 5 Symbolic interactionist theory

The major proponents of symbolic interactionism theory are George Herbert Mead, Charles Horton Cooley, W.I. Thomas, Herbert Blumer and Erving Goffman [29]. Herbert Blumer, American, a student, and a close friend of Mead, developed the term “symbolic interactionism” and advanced an influential summary of the perspective. According to Blumer, we act towards things based on the meaning that things have for us, and these meanings are derived from social interaction and modified through interpretation [29]. Symbolic interactions is a theory that approaches interaction or sees human interaction as emanating from interpretation of the meanings of signs and symbols and making a decision on how to react to these signs and symbols.

## 6 Weaknesses and application of symbolic interactionism to the utilization of maternal healthcare services and maternal mortality

To the symbolic interactionist, illness is socio-culturally defined. People may well have a serious disease, but this does not automatically result in people defining themselves as ill. This definition is a long process which involves social and cultural explanation. For instance Ref. [30], noted that in Nigeria and other West African countries, when a complication occurs in pregnancy, the decision of where to seek care depends on what is considered the cause of the complication. In the same view, women, especially rural women, often object to the use of obstetric and Gynecological services like cesarean operation, even when it seems the only alternative because of certain societal valves and expectations.

Thus, in the view of symbolic interactionism, the perception of illness stems from the complex mix between the individual and what they perceive to be good health, illness and the medical profession, and the views of the social network that surrounds the people [31]. Despite the usefulness of this theoretical approach in the analysis of health issues, it has not been without criticism. As [32] have noted, the social interactionism, account of health has been criticized for concentrating on the relationship between medical professionals and their patients while not paying attention to the wider social factors such as social inequality, stress etc which provide the actual causes of ill health.

## 7 Health Belief Model

Health Belief Model proposed by Hochbaum, Rosenstock & Kegels in 1950 is the commonly used framework in researches or studies which centers on health behaviour, the model offers useful recommendations towards prevention [33]. The model suggested; developed and modified to explain practices that can preventive ailments as well as contingent behaviour and the associated risks. It was developed by a group of social psychologists in the early 1950s [34] and simplified over a decade ago by Ref. [35]. Tinuola [36] proposed that a health belief model should function as a guide to the community on how to go through a preventive measures in taking the available health care delivery patterns.

## 8 Weaknesses and application of health belief model to the use of maternal healthcare services and maternal mortality

Health belief Model is a model which adopts a psychological approach to study and promote the uptake of services offered by a social psychologists, although subjected to various reviews by many scholars, it is important for health providers to access factors that inhibit the use of health seeking behaviour among pregnant women. The outcomes will stimulate further work on the choices and the interplay between knowledge and health behaviour. However, it is also the obligation of Cross River State government to institute an awareness campaigns to bring about a paradigm shift in orientation towards the use of maternal healthcare services among the populace which may improve on the therapeutic choices of the caregivers. It will serve as a guide for policy formulators on health towards the provision of adequate health care facilities for pregnant women.

This Model has received wide acceptability and application, most especially of recent, in predicting more general health behaviours. It offers a detail explanatory framework for understanding health behaviours. The extent and ability of identifying health problems, birth control, and risk behaviours depends on education and how knowledgeable pregnant mothers are in understanding these situations or phenomena. This depends on pregnant mothers’ exposure, attitude, orientation, personal disposition, personal enabling factors, and perceptions. Its framework is meant for motivating pregnant mothers to take positive health actions and decisions by using their discretion to avoid negative health outcomes. The Health Belief Model elaborates that preventive health behaviour is influenced by five factors of belief; which are the key variables, these are: Perceived Susceptibility to a health threat, Perceived Severity of the condition episode, Perceived Benefit to treatment, Perceived Barriers to consequences, and Cues to Action by caregivers.

**Perceived Susceptibility:** Means perception of people about getting a condition refers to the people perception of getting a condition, for instance, in reproductive health pregnancy, even when it is wanted or not [37]. discovered that mothers viewed their own vulnerability to the risk of the consequences of not utilizing maternal healthcare services. This is further interpreted to mean knowledge whether there is little or no risk of the consequences of their pregnancy, during pregnancy or after childbirth. However, if the individual does not think he/she is exposed to that condition, it can lead to a higher risk taking approach because there is no perception of susceptibility. The central thesis in the Health Belief Model is that when health situations are perceived to be serious, it leads to a higher implementation of preventive strategies. Research has always shown that health seeking behaviour is less for pregnant women and mothers who did not patronize maternal healthcare services, before, or after giving birth to their first child.

**Perceived Severity:** For every action there is a reaction which is an attribute of human behavioural pattern in the society. Every individual expects certain reactions as consequential to the initial action either from the immediate environment or the society at large [38]. perceived that severity on the other hand, has to do with one's belief on how serious a condition is. However, it focuses on different opinions on the severity of the medical, social, and financial consequences of any health behaviour. When pregnant mothers perceive pregnancy as a period that deserves adequate maternal medical care they will make patronize maternal healthcare services.

**Perceived Benefit:** This is the reward of the positive response that occurs from performing a recommended action. The action of a caregiver will largely be influenced by the perception that such an action will lead to reduction in the level of severity or vulnerability of pregnant mothers to maternal mortality. If a pregnant mother perceives that there are benefits derivable from utilizing maternal healthcare services, she is more likely to do so than another who is indifferent or does not see any benefit in acting.

**Perceived Barrier:** This dwells on how actions that are supposed to be taken n are not taken regardless of whether they believe that the benefits of taking such an action outweighs not taking action, this may be due to barriers [39]. Similarly argued that this assumption is based on the perception of the psychological, social, and financial costs of adopting a new health behaviours. Pregnant women will evaluate the potential barriers (physical, psychological, or financial) associated with the use of maternal healthcare services. This will influence their decisions on whether to seek an improvement in their health condition or not. However, the barriers could be psychological, financial, personal, enabling factor, personal disposition factor, and social.

**Self-Efficacy:** This explains the belief that one can successfully carry out the action required for producing the desired outcome and its influence as it affects health-related behaviour. Pregnant mothers will utilize maternal healthcare services because of the confidence that even if complications develop during childbirth or after child birth, the problem would have been foreseen and adequate preparations is made to tackle it. Positive steps must be taken for a person to remain healthy and the decision usually taken hang on three factors and these factors include; nature and pattern, human nature, and culture which have to do with health behaviour. For anybody to make health decisions, he must believe that he is at risk of that disease and that the degree of exposure may be either severe or mild [40].

**Cues to action:** It refers to an individual's perception of the levels of susceptibility and seriousness as being the factors that provides the force to act. Benefits (excluding barriers) will provide the first motivating factor towards the path to action. For instance, support for national policy as regards access to maternal healthcare facilities could even improve the choices of pregnant mothers in the utilization of maternal healthcare services.

## 9 Critical appraisal of the reviewed sociological theories

This section is devoted to a critical review or appraisal of all sociological and nonsociological theories that border on health and illness and other related theories that have been reviewed in this section. The purpose of this critical review is to simply recognize the advantages and disadvantages of the theories reviewed in this study with the aim of choosing the most appropriate of these theories for this work or study.

The first five theories reviewed include Consumer Behavior Theory [41,42], The biomedical perspective, The Protection-Motivation Theory [43], Self-regulatory Theory [44]. The five mentioned above emphasize health and illness and advance various reasons why healthcare should be utilized or the nonutilization of healthcare services. The combination of the above five theories will enable us to feel our way to the appropriate theoretical framework that should be adopted. The aspects of these five theories that is very relevant to this study emphasizes various reasons for the utilization or nonutilization of maternal healthcare services.

The aspects of these five theories that make it inadequate for this study is the emphasis on the biological, individualized, or healthcare providers, neglecting core cultural factors in the use or nonutilization of maternal healthcare services.

The theory of symbolic interactionism is equally relevant to this particular research or study. This is so because it gives us the insight into the explanations or reasons advanced for the occurrence of health and illness as varying from society to another. This position is supported by Ref. [45] that illness to a large extent is culturally determined.

Symbolic interactionisim and social cognitive theory are very relevant and important theories for this study. This is because both theories view illness, sickness, and disease as the way people think they are [46]. supported the above assertion when they postulated that the social context within which health and disease occur actually influence their definition.

The major pitfall of symbolic interactionism and its inadequacy to be used in this study is the limitation of maternal complications to social variables alone at the expense of other vital variables. There is a great wall of difference between maternal health complications defined via scientific means (objectives) and culturally defined (subjective), but the symbolic theory fails to emphasize or point out this difference which can be misleading.

[47] supported the above view when they asserted that the disease is not only ill health which is experienced consciously by the participants in a culture or sub-culture but also as one which is observed in a population with a competent diagnosis or analysis. According to Ref. [48], the criteria for confirming ill health should be bio-medical while the criteria for establishing illness are social and psychological.

A review of the social-cognitive theory traced the root cause of every human problem in society to the break down of law and order and the neglect of one or more basic societal needs. The theory emphasizes that a functioning society that has adequate norms and socialization process will guarantee a healthy individual within that society.

From the above, one can assert that this social cognitive approach is useful to this study because it avers that an inadequate socialization process coupled with the neglect of societal needs could result in maternal ill health in the society.

The major advantage of this theory and its relevance to this study, particularly with reference to health and illness, depends on the position that human problems generally have their causes located in the absence of one or more basic societal needs. For instance, social problems such as poverty, urban congestion, and unemployment could have some direct bearing on an individual state of well-being (health) and society in general.

## 10 Is the high incidence of maternal mortality due to poor access to health care services?

Literature is replete with statistics on the high incidence of maternal mortality caused by poor access to healthcare services. According to Refs. [16,48], about 99 % of maternal deaths occur in developing countries with poor access to healthcare services. Women resident in remote areas are more likely to face the challenge of poor access to health care services and skilled attendance because they are affected by poverty, distance, lack of information, inadequate service and cultural practices. At the various levels of the healthcare system, constraints to quality maternal healthcare services should be identified. In addition to setting global standards, provision of evidence based programmatic and clinical guidance and the provision of technical guidance to member states, the WHO in its bid to tackle poor access to health care services advocates the need for access to safe abortion practices, quality post abortion care and access to family planning even in remote areas. In a study on access to maternal healthcare services [22], submitted that 82 % of the respondents did not have quality access to maternal healthcare services, this finding is consistent with the results generated by Odutola et al. (2018), they presented findings which indicates that poor access to maternal healthcare services is intricately tied to maternal complications and mortality. Poor access to maternal healthcare services means poor monitoring during pre-natal, natal and post-natal stages of pregnancy.

Is the high incidence of maternal mortality due to poor access to health care services, or e.g., monitoring throughout pregnancy? Literature is replete with statistics on the high incidence of maternal mortality caused by poor access to healthcare services. According to Ref. [16], about 99 % of maternal deaths occur in developing countries with poor access to healthcare services. Women resident in remote areas are more likely to face the challenge of poor access to health care services and skilled attendance because they are affected by poverty, distance, lack of information, inadequate service and cultural practices. At the various levels of the healthcare system, constraints to quality maternal healthcare services should be identified. In addition to setting global standards, provision of evidence based programmatic and clinical guidance and the provision of technical guidance to member states, the WHO in its bid to tackle poor access to health care services advocates the need for access to safe abortion practices, quality post abortion care and access to family planning even in remote areas. In a study on access to maternal healthcare services [22], submitted that 82 % of the respondents did not have quality access to maternal healthcare services, this finding is consistent with the results generated by Ref. [48], they presented findings which indicates that poor access to maternal healthcare services is intricately tied to maternal complications and mortality. Poor access to maternal healthcare services means poor monitoring during pre-natal, natal and post-natal stages of pregnancy.

## 11 Conclusion

In spite of the above criticism, Health Belief Model and Symbolic Interactionism theories are relevant to this study, partly because, when combined, they view illness and patronage of healthcare facilities from both biological and social points of view. The perception of illness and the decision to visit hospitals are shaped by theoretical perception of illness, socio-cultural and economic implications of illness, and availability of health care services in Ugep. It's not enough to provide health facilities, the way the people define and act about illness equally influenced use of maternal health services.

## Conflict of interests

This article has no conflict of interest to report.

## Ethical considerations

This article was approved by Landmark University Ethical Board.

## Please state any conflicts of interest

There is no conflict of interest to report in this study.

## Please state any sources of funding for your research

There is no source of funding to report in this study. It was selffunded.

## Ethical Approval

This study did not involve any patient.

## Consent

The study did not involve patients or volunteers.

## Author statement

Ogadimma Arisukwu; Study concept, and writing of the paper.

Steven Akinfenwa; Data collection and literature review.

Chisaa Igbolekwu; Data analysis, and literature review.

## Registration of Research Studies

1 Name of the registry: Not applicable
2 Unique Identifying number or registration ID: Not applicable
3 Hyperlink to your specific registration (must be publicly accessible and will be checked): Not applicable

## Guarantor

Dr Ogadimma Arisukwu
